# SOCIAL NETWORK BARRIERS AND FACILITATORS TO RHEUMATOID ARTHRITIS MANAGEMENT IN HISPANIC/LATINX FAMILIES

**DOI:** 10.1093/geroni/igad104.2195

**Published:** 2023-12-21

**Authors:** Kamryn Wilson, Sara Dalpe, Zoe Waldman, Laura Koehly, Jielu Lin

**Affiliations:** National Human Genome Research Institute, Bethesda, Maryland, United States; National Human Genome Research Institute, Bethesda, Maryland, United States; National Human Genome Research Institute, Bethesda, Maryland, United States; National Human Genome Research Institute, Bethesda, Maryland, United States; National Human Genome Research Institute, Bethesda, Maryland, United States

## Abstract

Compared to their non-Hispanic White counterparts, individuals of Hispanic/Latinx heritage are more likely to develop disabilities from comorbidity and remain disabled for longer periods over the life course. Among chronic conditions, rheumatoid arthritis (RA) is very common, affecting at least 1% of the U.S. population. Evidence suggests that RA is more active and severe among Hispanic/Latinx individuals, characterized by more flare-ups, greater inflammation, higher tender/swollen joint counts, and more self-reported pain, due in part to their underuse of health services, delay in initiating treatment, and difficulty in adhering to treatment plan. In this study, we sought to identify social network factors relevant to managing RA pain and RA-attributable functional limitations, in a sample of Hispanic/Latinx families residing in the Washington DC region. We analyzed network structure and patterns of interpersonal health communication and assessed how these factors may facilitate or impede proactive healthcare, independent of individual and system-level barriers. The findings are three-fold: First, there is substantial heterogeneity in social network structure in the families examined, likely due to different immigration experiences. Second, severely contracted personal networks (e.g., isolate or singe-edge) were observed among older, foreign-born individuals who spoke Spanish and had less schooling. This was a major barrier to RA management. Finally, more health communication, closeness, and advice-seeking ties in one’s personal network facilitated use of health services which was positively correlated with health outcomes. We discuss the implications of the findings in the context of developing family network-based health education interventions.

